# A Maternal High Fat Diet Leads to Sex-Specific Programming of Mechanical Properties in Supraspinatus Tendons of Adult Rat Offspring

**DOI:** 10.3389/fnut.2021.729427

**Published:** 2021-09-13

**Authors:** Scott M. Bolam, Vidit V. Satokar, Subhajit Konar, Brendan Coleman, Andrew Paul Monk, Jillian Cornish, Jacob T. Munro, Mark H. Vickers, Benjamin B. Albert, David S. Musson

**Affiliations:** ^1^Bone and Joint Laboratory, University of Auckland, Auckland, New Zealand; ^2^Department of Orthopaedic Surgery, Auckland City Hospital, Auckland, New Zealand; ^3^Liggins Institute, University of Auckland, Auckland, New Zealand; ^4^Department of Orthopaedic Surgery, Middlemore Hospital, Auckland, New Zealand; ^5^Auckland Bioengineering Institute, University of Auckland, Auckland, New Zealand; ^6^Department of Nutrition, University of Auckland, Auckland, New Zealand

**Keywords:** tendon, animal model, rotator cuff, obesity, high-fat diet, developmental programming

## Abstract

**Background:** Over half of women of reproductive age are now overweight or obese. The impact of maternal high-fat diet (HFD) is emerging as an important factor in the development and health of musculoskeletal tissues in offspring, however there is a paucity of evidence examining its effects on tendon. Alterations in the early life environment during critical periods of tendon growth therefore have the potential to influence tendon health that cross the lifespan. We hypothesised that a maternal HFD would alter biomechanical, morphological and gene expression profiles of adult offspring rotator cuff tendon.

**Materials and Methods:** Female Sprague-Dawley rats were randomly assigned to either: control diet (CD; 10% kcal or 43 mg/g from fat) or HFD (45% kcal or 235 mg/g from fat) 14 days prior to mating and throughout pregnancy and lactation. Eight female and male offspring from each maternal diet group were weaned onto a standard chow diet and then culled at postnatal day 100 for tissue collection. Supraspinatus tendons were used for mechanical testing and histological assessment (cellularity, fibre organisation, nuclei shape) and tail tendons were collected for gene expression analysis.

**Results:** A maternal HFD increased the elasticity (Young's Modulus) in the supraspinatus tendon of male offspring. Female offspring tendon biomechanical properties were not affected by maternal HFD. Gene expression of SCX and COL1A1 were reduced in male and female offspring of maternal HFD, respectively. Despite this, tendon histological organisation were similar between maternal diet groups in both sexes.

**Conclusion:** An obesogenic diet during pregnancy increased tendon elasticity in male, but not female, offspring. This is the first study to demonstrate that maternal diet can modulate the biomechanical properties of offspring tendon. A maternal HFD may be an important factor in regulating adult offspring tendon homeostasis that may predispose offspring to developing tendinopathies and adverse tendon outcomes in later life.

## Introduction

Obese and overweight individuals make up over a third of the world's population and are projected to represent an estimated 60% by 2030 (1, 2). The impact of obesity on the musculoskeletal system is emerging as an important manifestation of the disease (3, 4). Several clinical and epidemiological studies have demonstrated a negative association between obesity and tendon health (5–7). In particular, obesity is consistently a risk factor for rotator cuff tendinopathy and tendon tear (5, 8–10). In animal models, a high fat diet (HFD) has been reported to result in detrimental effects on tendon biomechanical, structural and biochemical properties (11–13).

Over half of women of reproductive age are now overweight or obese (14, 15) and approximately one in five women are obese during pregnancy (16). Adverse maternal exposures during prenatal and early postnatal life can impair offspring health across their lifespan. Maternal obesity is a risk factor for increased adiposity and glucose intolerance in adult offspring (17). In rodent pre-clinical studies, maternal obesity can be modelled using a HFD through well-established methods that has been previously demonstrate to alter maternal and offspring metabolic profiles (18–20).

A maternal HFD has a significant influence on the development and health of other musculoskeletal tissues in offspring, including bone, cartilage and muscle (21–25). Despite the increasing global prevalence of the high-fat Western diet, the effect of maternal exposure to HFD on programming of offspring tendon health is largely unknown. Alterations in the early life during critical periods of tendon development have the potential to influence offspring tendon gene, cell and tissue function throughout adult life.

The purpose of this study was to determine the effect of a maternal HFD on the tendon profile of adult male and female offspring. We hypothesised that a maternal HFD would be associated with altered biomechanical and structural properties in offspring tendon, and altered gene expression profiles of offspring tendon cells.

## Materials and Methods

### Animal Model

All animal experiments were approved by the University of Auckland Animal Ethics Committee (#001936), in accordance with the New Zealand Animal Welfare Act, 1999. Reporting of *in vivo* results conforms to the ARRIVE guidelines (18). This study of tendon properties was conducted on a subset of adult offspring (*n* = 8 per group) from an unrelated larger study investigating metabolic end points in adult offspring with dietary manipulation and fish oil supplementation of pregnant rat dams (*n* = 12 per group).

**Figure 1:**
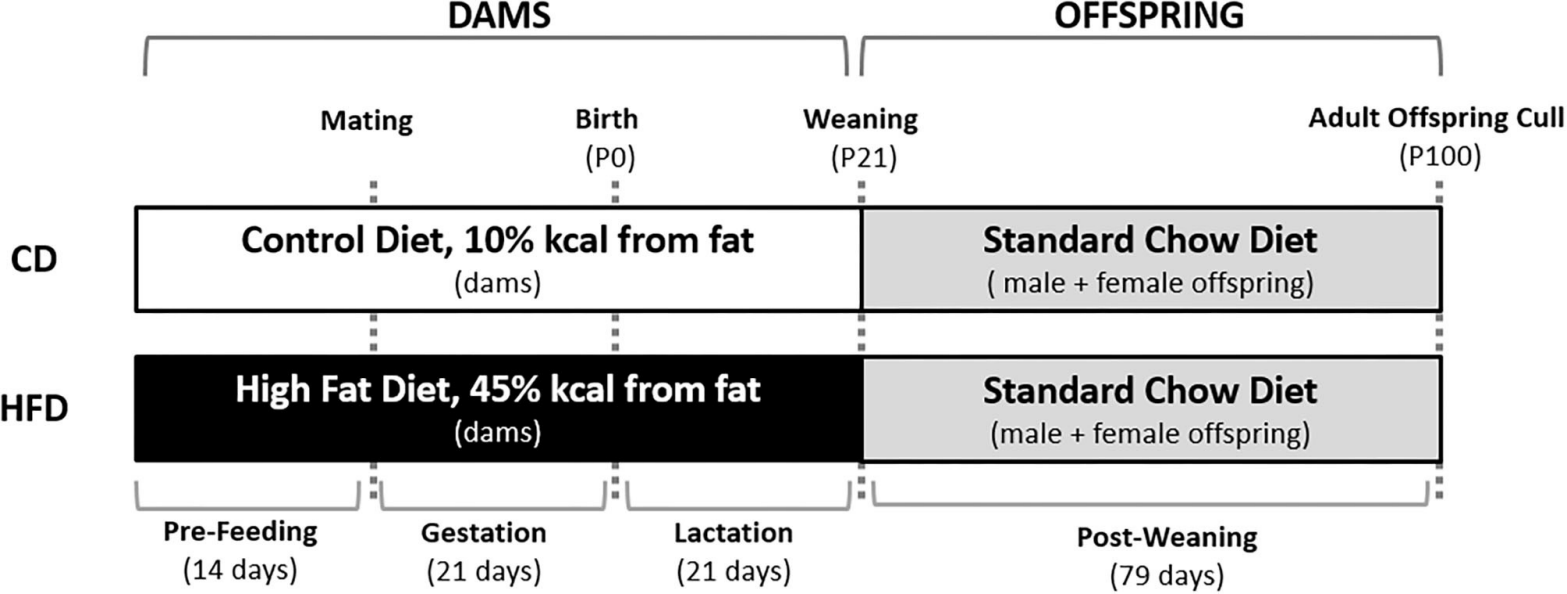
Overview of the study design. Female Sprague-Dawley rats consumed either a CD or HFD *ad libitum* for 14 days prior to mating and throughout pregnancy and lactation. Male and female offspring were weaned onto a standard chow diet *ad libitum* at post-natal day 21 (P21). At post-natal day 100 (P100), offspring were culled and plasma and tissue was collected.

Female virgin Sprague-Dawley rats were obtained at a weaning age and fed a standard chow diet (Diet 2018, Envigo Teklad Global Diets, Indianopolis, IN, USA) *ad libitum* until day 110. All animals were housed under standardised conditions at 22 ± 2°C with a 12 h light: 12 h dark cycle. At day 110, rats were then randomly assigned to either a control diet (CD; 10% kcal or 43 mg/g from fat, #D12450H, Research Diets, Diets, New Brunswick, NJ, USA) or HFD (45% kcal or 235 mg/g from fat, #D12451, Research Diets) and fed *ad libitum* for 14 days prior to mating and throughout gestation and lactation. Females were time mated using an oestrous cycle monitor (EC-40, Fine Science Tools, San Francisco, CA, USA), with pregnancy confirmed through detection of spermatozoa following vaginal lavage. Dams were continued on their study diet throughout the pregnancy and lactation (Figure 1).

In order to maintain standardised nutrition until weaning, litter size was randomly adjusted to eight pups per litter (4 males and 4 females) on post-natal day 2 (P2) and unused pups were euthanized. At P21, one male and one female offspring from each dam were randomly selected. Offspring were housed in sex-matched pairs and fed a standard chow diet *ad libitum* for the remainder of the study (P100). Body weights were monitored every third day. At P100, animals were fasted overnight and killed by decapitation under pentobarbitone anaesthesia (Sigma-Aldrich, St Louis, MO, USA; intraperitoneal injection; 60 mg/kg). Eight male and eight female offspring were randomly selected from each of the maternal dietary groups for tendon analysis.

From these offspring, both shoulders with the supraspinatus tendon attached to the humerus were excised immediately. The left shoulders were wrapped in a phosphate-buffered saline solution-soaked gauze and stored at −20°C for later biomechanical testing and the right shoulders were immersed in 10% neutral buffered formalin (NBF) for histological analysis. The tail was harvested and primary tenocytes were isolated from tendon fascicles for gene expression analysis. Trunk blood was collected into heparinised vacutainers (Becton Dickinson, Franklin Lakes, NJ, USA) and centrifuged at 2,500 g at 4°C for 15 min. Plasma samples were stored at −20°C until subsequent analysis.

### Plasma Analysis

Plasma was analysed for free fatty acids, triglycerides, low-density lipoprotein cholesterol (LDL), high-density lipoprotein cholesterol (HDL) and total cholesterol by Hitachi 902 auto-analyser (Hitachi High Technologies Corporation, Tokyo, Japan). Plasma samples were thawed on ice and centrifuged for 3 min at 2,500 rpm at 4°C prior to performing the kits to remove fibrous clots which are common in rodent plasma. Where multiple plates were needed for one marker, samples were performed in a randomised order which was generated via an Excel database to avoid time-of-day effects and inter-assay effects. All intra- and inter- assay coefficients of variation were <5%. Plasma analysis was performed with 8 animals per group.

### Biomechanical Testing

Biomechanical testing was performed using established protocols, as previously described (26). The excised shoulders were thawed at room temperature and kept hydrated with normal saline solution spray throughout testing. The supraspinatus muscle fibres were removed by gentle scraping, leaving only the distal tendon attached to the proximal humerus. The humerus and the tendon were positioned in an Instron machine (Instron, Norwood, MA, USA) with a 1-kN load cell. The tendon was secured using a double screw clamp with fine-grit sand paper, and the humerus was secured using a customised 3D printed clamp to prevent fracture through the growth plate. The width and thickness of the tendon were measured using digital callipers and cross-sectional area calculated by multiplying both measurements. The specimens were subjected to uniaxial strain in line with anatomical loading and undergo a 10-cyle precondition (0.1 to 0.5 N at rate of 0.5 mm/sec) followed by 1 min of relaxation and were then stretched to failure at a rate of 0.5 mm/s. The Young's modulus and ultimate stress at failure were recorded for each sample. Specimens mostly failed at the bone-tendon interface of the supraspinatus enthesis and only these results were included. In the three other instances, failure occurred prematurely intra-substance in the supraspinatus tendon. Therefore, 5 or 6 samples were tested for each group.

### Histological Analysis

After fixing in 10% NBF for 7 days, bone-tendon-muscle units were decalcified in formic acid 10% aqueous solution for 7 days and then processed, embedded in paraffin and 7-μm-thick sections were taken. Sections were stained with haematoxylin and eosin and the tendon mid-substance was imaged at 40x magnification and viewed using both transmitted and polarised light. Cell density (number of nuclei per mm^2^) and nuclear aspect ratio (the ratio of the minor diameter to the maximal diameter, with values approaching zero suggesting a spindle shape and with the value of 1.00 representing a perfect circle) were evaluated, as described in previously established protocols (27, 28). In healthy tendon, the few fibroblasts with flattened nuclei are typically aligned parallel to the tensile axis. In tendinopathy, tendon cells density increases, nuclei become more rounded, and collagen fibre alignment is disrupted (27). Six regions of interest (ROI) were measured and the mean value was taken for each sample.

The Directionality plug-in for Fiji (http://fiji.sc/Fiji, Ashburn, VA, USA) was used on polarised light images to perform 2D fast Fourier transform analysis to measure collagen fibre alignment, according to previously established methods (29, 30). The Directionality plug-in calculated the spatial frequencies within an image given a set of radial directions. The method generated normalised histograms revealing the amount of fibres present between 0° and 180° with a bin size of 1°. The plug-in then generated statistics on the highest peak found and performed a Goodness-of-fit test between the observed values and a Gaussian distribution to provide a Goodness value [0 (poorly aligned) -1 (well-aligned)]. The Goodness values were measured in six ROI's and the mean value was taken for each sample. Six samples were analysed for each group.

### Gene Expression Analysis of Tenocytes Isolated From Rat Tail Tendons

Tenocytes were isolated from tendon fascicles teased from rat tails using a previously established protocol (31). In brief, tendon was cut into <1 cm pieces and digested in 0.5 mg/ml dispase and 400 U/ml collagenase-II (both from Sigma-Aldrich, St. Louis, MO, USA) in Dulbecco's Modified Eagle's Medium: F-12 with 10% FBS at 37°C for up to 18 h until most of the extracellular matrix had been digested. The cell suspension was then passed through a cell strainer, washed, re-suspended in phosphate-buffered saline and pelleted for RNA extraction.

For analysis of gene expression, total cellular RNA was extracted from cultured cells and purified using the RNeasy minikit (Qiagen, Venlo, The Netherlands). Genomic DNA was removed using RNasefree DNase set (Qiagen). Quality and concentration of the extracted RNA was measured using NanoDrop Lite Spectrometer (Thermo-Fisher, Victoria, Australia). Complementary-DNA (cDNA) was prepared by using 300 ng of RNA with super-script-III (Life Technologies, Carlsbad, CA, USA). Primer-probe sets were purchased as TaqMan Gene Expression Assays (Life Technologies). Multiplex polymerase chain reaction was performed with FAM specific for genes of interest and VIC-labelled 18S endogenous ribosomal RNA probes, according to the manufacturer's instructions, using an ABI PRISM 7900HT sequence detection system (Applied Biosystems, Foster City, CA, USA). Samples were assayed in triplicate. The ΔΔCt calculation method was used to determine the relative level of messenger RNA expression, normalised to the values of cells from a CD offspring for each sex. The relative gene expression of collagen type Iα1 (COL1A1), the main structural collagen present in tendon (32); scleraxis (SCX), a key transcription factor in tenocyte differentiation (33); tendomodulin (TNMD), a key glycoprotein in the proliferation and development of tenocytes (34), were determined. In addition, chondrocyte gene, SOX-9, was assessed as tenocytes tend to transdifferentiate into such cell types, one of the pathological causes of tendinopathy (35). Matrix re-modelling [matrix metalloproteinase-3 (MMP-3) and MMP-13] gene expression markers were also determined as the basal activity of MMPs is greatly modified in painful tendinopathy (36). Four samples *per sex* were analysed for each group.

### Statistical Analysis

Data from the weights, blood samples, histological, biomechanical and gene expression were analysed using a Mann-Whitney *U*-test for comparison between maternal CD and maternal HFD in both male and female offspring. *P* < 0.05 was considered significant. Data are presented as median with interquartile range (IQR) and graphed using Prism 8 software (GraphPad Software, Inc., La Jolla, CA, USA).

## Results

### Maternal HFD Did Not Induce an Overt Change in Dam Body Weight and Offspring Body Weight or Metabolic Profile

At P21, there were no significant differences in the weights of dams fed a CD or HFD diet. There were no significant effects of a maternal HFD on adult offspring weights at P100 for either sex. In addition, there were no significant differences in free fatty acids, triglycerides, HDL, LDL and total cholesterol in either male or female offspring with maternal HFD (Table 1).

**Table 1:** Physiological and metabolic profiles of each group.

	**Male offspring**			**Female offspring**		
**Maternal Diet**	**CD**	**HFD**	***P*-Value**	**CD**	**HFD**	***P*-Value**
Dam weight P100	314.8 (19.0)	320.8 (52.4)	0.867	314.8 (19.0)	320.8 (52.4)	0.867
Offspring weight P100 (g)	465.5 (26.6)	511.1 (66.6)	0.142	259.6 (72.0)	274.8 (61.2)	0.220
Free fatty acids (mmol/L)	0.54 (0.09)	0.67 (0.35)	0.512	0.55 (0.23)	0.66 (0.15)	0.067
Triglycerides (mmol/L)	0.69 (0.41)	0.73 (0.49)	0.662	0.74 (0.22)	0.60 (0.36)	0.377
HDL (mmol/L)	1.49 (0.66)	1.17 (0.43)	0.363	1.80 (0.45)	1.80 (0.50)	0.804
LDL (mmol/L)	0.48 (0.17)	0.37 (0.19)	0.216	0.29 (0.18)	0.28 (0.15)	0.980
Total cholesterol (mmol/L)	2.10 (0.86)	2.26 (0.70)	0.662	1.73 (0.87)	2.24 (0.70)	0.557

*Data were analysed by Mann-Whitney U-test for each sex. Data are presented as median (with IQR). N = 8 per group*.

### A Maternal HFD Impacted Tendon Elasticity in Adult Male Offspring, but Did Not Affect Tendon Biomechanical Properties in Female Offspring

In male offspring, the Young's modulus was significantly higher with maternal HFD compared to maternal CD [29.72 (IQR 28.94) vs. 59.23 (IQR 15.85), *P* = 0.030]. There was no significant effect of maternal HFD in female offspring [37.71 (IQR 24.17) vs. 41.78 (IQR 22.62), *P* = 0.222]. There was no significant difference in stress of failure and cross-sectional area between HFD CD offspring groups (Table 2).

**Table 2:** Supraspinatus tendon biomechanical properties.

	**Male offspring**			**Female offspring**		
**Maternal Diet**	**CD**	**HFD**	***P*-Value**	**CD**	**HFD**	***P*-Value**
Cross-sectional area (cm^2^)	1.17 (0.04)	1.16 (0.25)	0.695	1.08 (0.16)	1.30 (0.26)	0.159
Young's modulus (MPa)	29.72 (28.94)	59.23 (15.85)^*^	**0.030**	37.71 (24.17)	41.78 (22.62)	0.222
Stress at failure (MPa)	20.52 (13.33)	27.54 (7.40)	0.429	16.71 (5.26)	19.55 (11.84)	0.222

*Data were analysed by Mann-Whitney U-test for each sex, where*

* *P < 0.05 compared to CD offspring. Data are presented as median (with IQR). N = 6 per group.*

*Values in bold are significantly different (P < 0.05) compared to maternal CD*.

### Offspring Tendon Histological Structure Was Not Affected With Maternal HFD

**Figure 2:**
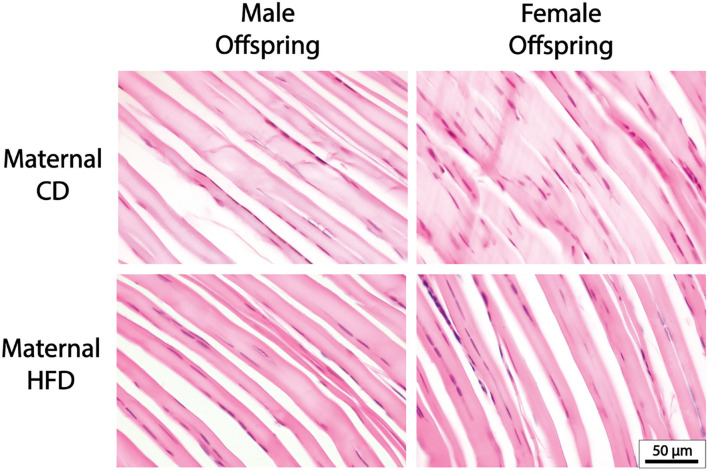
Representative Tendon Histology Images. There were no significant differences in cellularity, collagen alignment or nuclei shape with maternal HFD in either male or female offspring.

**Figure 3:**
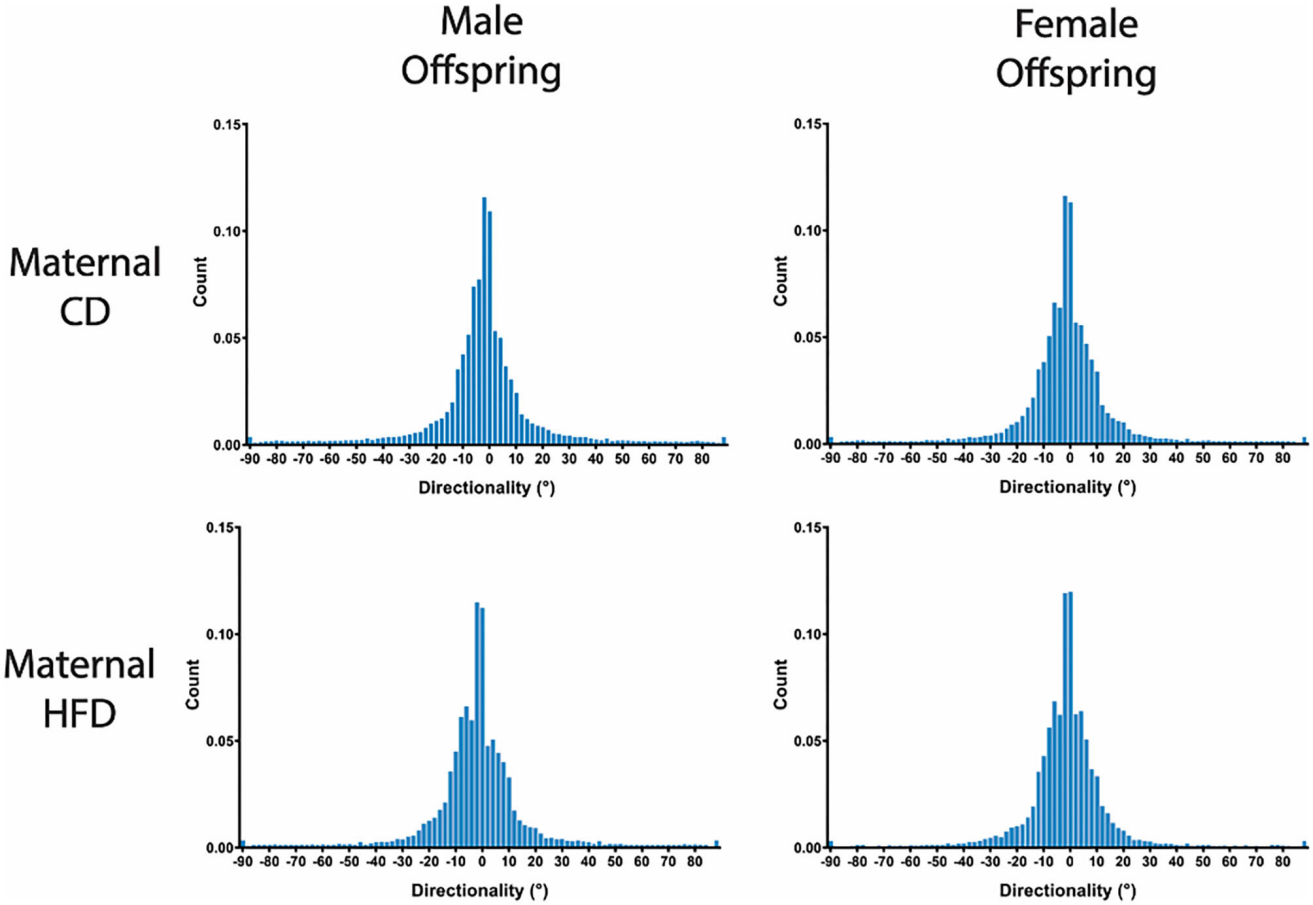
Group averaged Directionality Histograms of Collagen Fibre Alignment. Averaged histograms demonstrating the distribution of collagen fibres present between 0° and 180° with a bin size of 1° for each sex and maternal diet. *N* = 6 per group.

A maternal HFD had no significant effect on cell density or nuclei shape in either male or female offspring, compared to maternal CD (Table 3; Figure 2). A maternal HFD also did not significantly affect tendon collagen organisation (Directionality Goodness value) in either sex (Table 3). Averaged normalised histograms demonstrating the similar distribution of collagen fibre alignment for each sex and maternal diet are shown in Figure 3.

**Table 3:** Detailed histologic scores.

	**Male offspring**			**Female offspring**		
**Maternal Diet**	**CD**	**HFD**	***P*-Value**	**CD**	**HFD**	***P*-Value**
Cellularity (cells per mm^2^)	1,101 (329.7)	1,067 (326.5)	0.937	1,413 (336.0)	1,359 (301.0)	0.574
Nuclei Circularity (0–1)	0.425 (0.236)	0.497 (0.182)	0.485	0.524 (0.061)	0.476 (0.149)	0.126
Directionality Goodness Value (0–1)	0.926 (0.056)	0.883 (0.073)	0.071	0.920 (0.073)	0.914 (0.050)	0.662

*Data were analysed by Mann-Whitney U-test for each sex. Data are presented as median (with IQR). N = 6 per group*.

### Lower Gene Expression of Scleraxis in Male Offspring and Collagen Type Iα1 in Female Offspring of Dams Fed a HFD

**Figure 4:**
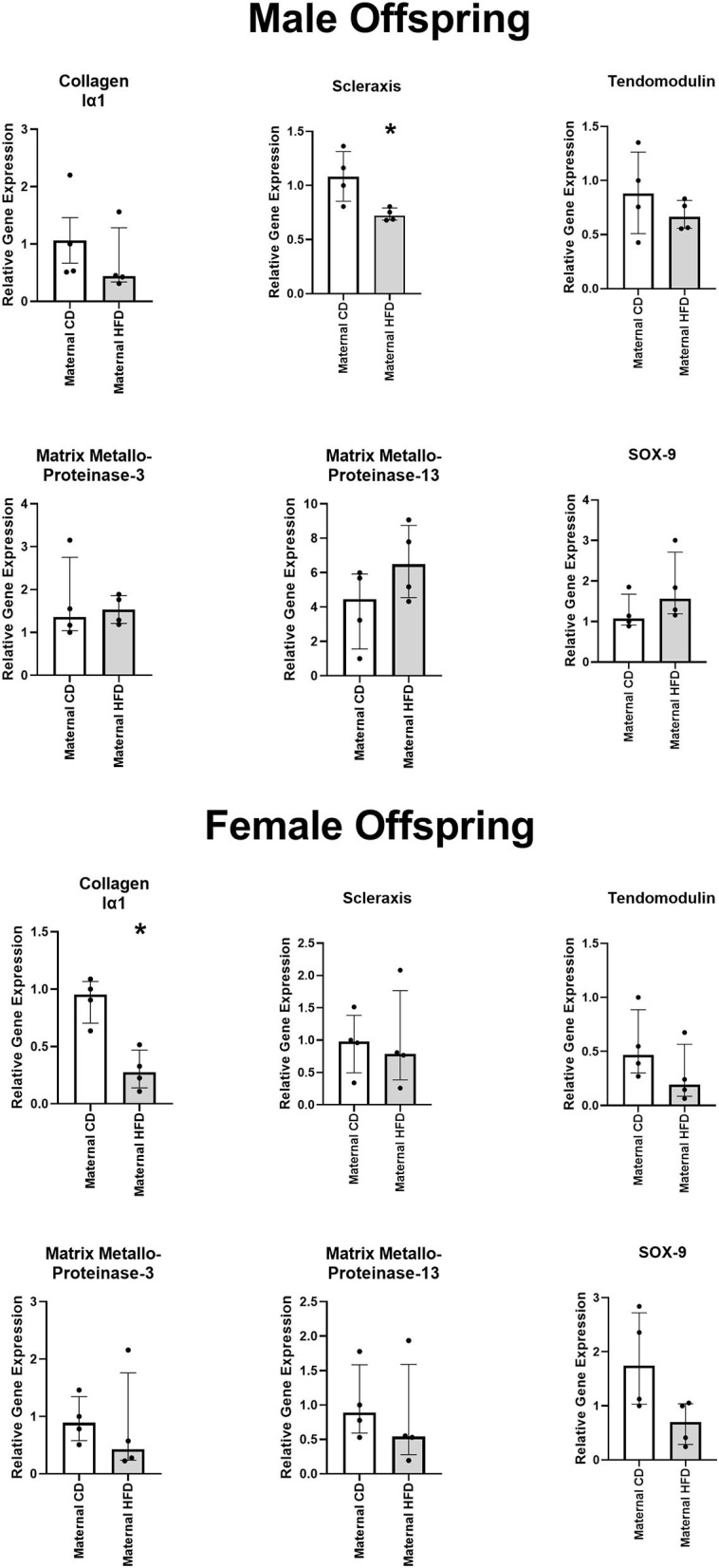
Effect of maternal HFD on the gene expression profile of primary tenocytes derived from tail tendon. Data were analysed by Mann-Whitney *U*-test for each sex. Data are presented as median (with IQR), where *P < 0.05 compared to CD offspring. *N* = 4 per group.

In primary tenocytes of the male, but not female, offspring of maternal HFD, there was significantly lower expression of tenocytic marker SCX gene (−33%, *P* = 0.029). In female, but not male, offspring of maternal HFD there was lower expression of tenocytic COL1A1 gene (−71%, *P* = 0.029) (Figure 4). There was a strong trend in reduction of chondrocytic maker SOX-9 in female offspring of dams fed HFD, however this did not reach statistical significant (*P* = 0.061). Maternal diet had no significant effect on the gene expression levels for other tenocyctic (TNMD), chrondrocytic (SOX-9) or matrix re-modelling (MMP-3 and MMP-13) markers in either male or female offspring.

## Discussion

Here, we have demonstrated for the first time that maternal diet can influence offspring tendons. A maternal diet high in fat increased the Young's modulus of the supraspinatus tendon of male offspring, and altered the gene expression profile of tendon cells from offspring of both sexes. However, maternal HFD did not significantly alter the histological structural properties of supraspinatus tendon in male or female offspring.

A maternal obesogenic diet has been associated with a range of adverse effects across several physiological systems (37–39). Emerging pre-clinical and clinical evidence suggests that maternal exposure to HFD may have long-term consequences on the musculoskeletal system in offspring (40–45). A maternal HFD has been shown to increase the size of type 1 and 2A fibres in skeletal muscle, promoting a more oxidative profile, and elicit lifelong mitochondrial alterations (46–48). In bone, maternal HFD has been demonstrated to negatively impact osteoblast performance and lead to osteopenia in adult offspring, independent of post-weaning diet (44, 45). Foetal pre-osteoblastic cell senescence signalling appears to be epigenetically regulated by maternal obesity to repress bone formation in adult offspring (23).

Previous studies have reported direct detrimental effects of consuming a HFD on the biomechanical properties of rodent tendon (11–13). Interestingly, these tendinopathic changes do not seem to resolve with dietary intervention from a HFD to low-fat diet, suggesting that any pathologic change induced in tendon with HFD is irreversible (49). Previous studies of other musculoskeletal tissues, including muscle and bone, have shown that post-weaning exposure to HFD has an additive deleterious effect with maternal HFD exposure on programmed diseased tissue phenotypes (23, 47). We therefore may have seen more pronounced effects on tendon properties with exposure to a post-weaning HFD in offspring. Future studies should investigate the possible additive effects of maternal HFD and the offspring ageing on adverse tendon outcomes.

We found sex-specific effects of a maternal HFD in alteration of offspring tendon properties, with only male tendon elasticity reduced at P100. Sex-specific variability in tendon biomechanical properties is well-established and thought to be the result of hormonal influence (50–53). Elevated levels of oestradiol reduce collagen synthesis in ligaments and tendons, and thus collagen remodelling may be less pronounced in females. This could be reducing the responsiveness of female offspring tendon to the effects of maternal HFD (52, 53). Another possibility is that sex-specific differences in the underlying epigenetic regulation of genes associated with tendon development could be contributing to this variation.

In this study there was lower expression of tenocytic-marker SCX gene in male, but not female, offspring of a maternal HFD. Scleraxis is a basic helix-loop-helix transcription factor and plays a central role during embryonic tendon development. SCX gene expression drives matrix production and re-modelling, epithelial-to-mesenchymal transition, development of force-transmitting tendon and tendon growth (54–57). Scleraxis is required for mechanically stimulated adult tendon growth through driving the expression of extracellular matrix (ECM) components, so changes in SCX expression could have diverse impact on offspring tendon development (56, 57). In female, but not male, offspring of a maternal HFD, there was reduced of expression of type I collagen gene. Collagen type Iα1 accounts for the majority of dry weight of tendon ECM. A major focus of tenocyte metabolism in post-natal tendon tissue is to maintain the ECM integrity by regulating type I collagen production (58, 59). This is the first evidence that maternal HFD can influence gene expression in adult offspring and requires more research to determine the regulatory pathways which may be altering their expression.

Determining the mechanisms by which a maternal HFD diet results in altered tendon properties will require additional study. Previous studies have suggested that effect of obesity on tendon may be secondary to alterations with circulating cholesterol and lipid levels (11, 13). It has been proposed that elevated cholesterol levels may alter the tendon microenvironment via local changes in protein synthesis and extracellular matrix composition/turnover (60). In this study, there were no differences in the lipid and cholesterol profiles of offspring animals with a maternal HFD. This suggests that effects on offspring tendon are occurring independently of hypercholesterolemia. There is a growing understanding of epigenetic control in the onset and progression of other musculoskeletal diseases, including osteoarthritis and osteoporosis (25, 61, 62). There is evidence of epigenetic regulation in the expression of genes specifically associated with tendon development, including scleraxis, collagen type Iα1 and tendomodulin (63). Other studies have also shown that maternal and paternal epigenetic modifications play essential roles in the development of tendon (64–66). Thus, we speculate the maternal diet could alter epigenetic histone modifications, DNA methylation, and non-coding RNAs in offspring to confer susceptibility to tendinopathy and tendon rupture that persists throughout life.

## Limitations

The rats in this experiment were a relatively young age of 14 weeks old (P100) when tissue was collected. Ageing is a known risk factor in the onset of tendinopathy and therefore a longer study period may have resulted in more significant changes in tendon properties. Similarly, more significant changes may have been observed following injury to the tendons, with a maternal HFD potentially predisposing the tendons to poorer healing outcomes. Therefore, future research could consider not only the effects of maternal diet on tendon health, but also tendon injury and healing.

In this study we only included a sub-set of animals from a large study for assessment of tendon properties and it is possible higher animal numbers may have identified further differences. However, the sample size chosen for tendon analysis was based on previous experimental studies (*n* = 6–9 per group) investigating the effect of post-weaning HFD exposure on biomechanical and histological properties (11, 12, 67).

Here, we used supraspinatus tendon to explore the biomechanical and histological effects of maternal HFD, and rat tail tendon to explore changes in tenocyte gene expression profiles. It would have been optimal to explore all outcomes from the same tendon origin, as this would allow for associations to be made between the gene and tissue level changes. However, the supraspinatus tendon is not suitable for obtaining RNA/cells to carry out such studies due to its small size and acellular nature. The rat tail, however, is a well-validated source of tenocytes and represents a good model of tendon cell behaviour (68–72). While we were unable to correlate gene level changes with tissue level alterations, individually these findings are novel and provide valuable information about the effect of maternal high fat diet on offspring tendon properties.

Furthermore, changes in tenocyte behaviour were determined at the gene expression level from RNA extracted from cell pellets digested direct from the tail tendon fascicles. The whole cell pellet was used for this purpose, to ensure there was sufficient extracted RNA. This prevented the gene expression changes observed being validated with corresponding protein level analysis, which will be important to look at in future studies.

The length of maternal exposure to HFD was relatively short in this study, and although it included the entirety of pregnancy and lactation, the dietary intervention began only 14 days prior to mating. We may have observed more pronounced effects on offspring tendon phenotype with longer duration of pre-conception maternal HFD. The lack of an overt programmed metabolic phenotype in our offspring may also be a result of the control diet utilised. The current study utilised a matched semi-purified control diet in the dams, previous work has utilised a standard chow-based diet and the differences in energy/caloric intake between these control diets may explain the differences in phenotypes observed (18). Programming effects in offspring can be subtle when fed a standard diet post-natally and only amplified in the setting of a post-weaning HFD (23, 47). Thus, further studies could examine the effects of maternal HFD alone and combination with a postnatal HFD in offspring.

Finally, this study was conducted in a rodent model, and although maternal HFD during pregnancy is a well-established model of offspring obesity independent of post-natal diet, there are discrepancies in gene expression alterations between obese rats and humans which could potentially limit the applicability to humans (73).

## Conclusions

This is the first study to demonstrate that maternal diet influences tendon homeostasis and biomechanical properties in adult offspring. This research suggests that maternal HFD may be an important factor in regulating an offspring tendon phenotype that predisposes adult offspring to adverse tendon outcomes and higher prevalence of tendon injury in adult life.

## Data Availability Statement

The original contributions presented in the study are included in the article/supplementary material, further inquiries can be directed to the corresponding author.

## Ethics Statement

The animal study was reviewed and approved by University of Auckland Animal Ethics Committee.

## Author Contributions

SB participated in study design, acquisition of data, interpretation of data, drafting of the article, and revision of the manuscript. VS participated in study design, acquisition of data, interpretation of data, and revision of the manuscript. SK participated in acquisition of data, drafting of the article, and revision of the manuscript. BC, AM, and JC participated in analysis, interpretation of data, and revision of the manuscript. JM participated in study design, analysis, interpretation of data, and revision of the manuscript. MV participated in study design, acquisition of data, analysis, interpretation of data, and revision of the manuscript. BA participated in study design, acquisition of data, analysis, and revision of the manuscript. DM participated in study design, acquisition of data, analysis, drafting of the article, and revision of the manuscript. All authors contributed to the article and approved the submitted version.

## Funding

This research was supported by the Auckland Medical Research Foundation #1117017 project grant and senior research fellowship (DM) and the Health Research Council of New Zealand #20/027 (SM) as well as an Australasian Paediatric Endocrine Care research grant #3714179 (BA).

## Conflict of Interest

The authors declare that the research was conducted in the absence of any commercial or financial relationships that could be construed as a potential conflict of interest.

## Publisher's Note

All claims expressed in this article are solely those of the authors and do not necessarily represent those of their affiliated organizations, or those of the publisher, the editors and the reviewers. Any product that may be evaluated in this article, or claim that may be made by its manufacturer, is not guaranteed or endorsed by the publisher.

## Abbreviations

ARRIVE: Animal research: reporting of *in vivo* experiments
CD: Control diet
cDNA: Complementary DNA
COL1A1: Collagen type I alpha 1 chain
DNA: Deoxyribonucleic acid
ECM: Extracellular matrix
HFD: High fat diet
IQR: Interquartile Range
MMP: Matrix metalloproteinase
RNA: Ribonucleic acid
ROI: Regions of interest
SCX: Scleraxis
SEM: Standard error of the mean
TNMD: Tendomodulin
2D: Two dimensional
3D: Three dimensional.
